# Transglutaminase 2, a Novel Regulator of Eicosanoid Production in Asthma Revealed by Genome-Wide Expression Profiling of Distinct Asthma Phenotypes

**DOI:** 10.1371/journal.pone.0008583

**Published:** 2010-01-05

**Authors:** Teal S. Hallstrand, Mark M. Wurfel, Ying Lai, Zhanglin Ni, Michael H. Gelb, William A. Altemeier, Richard P. Beyer, Moira L. Aitken, William R. Henderson

**Affiliations:** 1 Division of Pulmonary and Critical Care, Department of Medicine, University of Washington, Seattle, Washington, United States of America; 2 Division of Allergy and Infectious Diseases, Department of Medicine, University of Washington, Seattle, Washington, United States of America; 3 Department of Environmental Health, University of Washington, Seattle, Washington, United States of America; 4 Department of Chemistry and Biochemistry, University of Washington, Seattle, Washington, United States of America; University of Giessen Lung Center, Germany

## Abstract

**Background:**

A frequent manifestation of asthma, exercise-induced bronchoconstriction (EIB), occurs in 30–50% of asthmatics and is characterized by increased release of inflammatory eicosanoids. The objective of this study was to identify genes differentially expressed in EIB and to understand the function of these genes in the biology of asthma.

**Methodology/Principal Findings:**

Genome-wide expression profiling of airway leukocytes and epithelial cells obtained by induced sputum was conducted in two groups of subjects with asthma with and without EIB (n = 7 per group), at baseline and following exercise challenge. Based on the results of the gene expression study, additional comparisons were made with a normal control group (n = 10). Localization studies were conducted on epithelial brushings and biopsies from an additional group of asthmatics with EIB (n = 3). Genes related to epithelial repair and mast cell infiltration including β-tryptase and carboxypeptidase A3 were upregulated by exercise challenge in the asthma group with EIB. A gene novel to asthma pathogenesis, transglutaminase 2 (TGM2), was the most differentially expressed at baseline between the groups. *In vivo* studies confirmed the increased expression of TGM2 in airway cells and airway lining fluid, and demonstrate that TGM2 is avidly expressed in the asthmatic airway epithelium. *In vitro* studies using recombinant human enzymes reveal that TGM2 augments the enzymatic activity of secreted phospholipase A_2_ (PLA_2_) group X (sPLA_2_-X), an enzyme recently implicated in asthma pathogenesis.

**Conclusions/Significance:**

This study found that TGM2, a mediator that is novel to asthma pathogenesis, is overexpressed in asthmatic airways and functions to increase sPLA_2_-X enzymatic activity. Since PLA_2_ serves as the first rate-limiting step leading to eicosanoid formation, these results suggest that TGM2 may be a key initiator of the airway inflammatory cascade in asthma.

## Introduction

Although asthma is the most common chronic disease among young adults, the molecular basis of asthma, especially related to non-allergic stimuli remains poorly understood. A fundamental barrier to understanding asthma is the heterogeneity of the phenotype and characteristic pattern of inflammation in the lower airways. One frequent manifestation of asthma is exercise-induced bronchoconstriction (EIB), a syndrome in which a brief period of exercise triggers airway narrowing lasting 30–90 min [1]. Cross-sectional studies demonstrate that EIB occurs in a distinct group of subjects with asthma, representing about 30–50% of all asthmatics [2], [3], [4], [5], [6]. The severity of EIB is associated with other manifestations of indirect airway hyperresponsiveness (AHR) to hypertonic aerosols and adenosine [7].

We have previously demonstrated that there is a distinct pattern of airway inflammation in asthmatics with EIB that is notable for increased concentrations of inflammatory eicosanoids such as cysteinyl leukotriene (CysLT)s and disrupted lower airway epithelial cells in induced sputum [8]. Increased concentrations of CysLTs have also been identified in exhaled breath condensate of children with EIB [9]. We and others have shown that these eicosanoids along with cellular products from mast cells and eosinophils have a pivotal role in the pathogenesis of EIB, causing airway narrowing and mucin release [10], [11]. Inhibitors of mast cell activation and the effects of mast cell products such as histamine and CysLTs reduced the severity of EIB [12], [13]. A key regulator of eicosanoid release, secreted phospholipase A_2_ group X (sPLA_2_-X), was recently shown to be increased in the airways of asthmatics, and further increased after exercise challenge [14]. Deletion of the sPLA_2_-X gene in a murine model of asthma inhibits development of airway inflammation, AHR, and structural remodeling [15].

Based on the distinct phenotypic characteristics and immunopathology of EIB, we hypothesized that there are distinct molecular pathways transcriptionally active in the airways of asthmatics with EIB. To address this hypothesis, we identified two groups of asthmatics with AHR to methacholine that were discordant for severity of EIB, and compared gene expression in airway leukocytes and epithelial cells in induced sputum between the groups at baseline and then on a separate day following exercise challenge. To assure that the results were reproducible, we enrolled the subjects into initial and replication cohorts to identify genes with consistent differences in gene expression, and used a second method to identify differences in gene expression based on the false discovery rate. Based on differences in gene expression, we confirmed that transglutaminase 2 (TGM2) was over-expressed in airway cells by quantitative PCR (qPCR), and that the TGM2 protein is elevated in the airway lining fluid of asthmatics with EIB relative to the asthma group without EIB. Additional studies compared the expression and protein levels between asthmatics and non-asthmatic controls, and defined the distribution of TGM2 in the asthmatic airway epithelium. *In vitro* studies revealed that recombinant human TGM2 causes a sustained increase in the enzyme activity of recombinant human sPLA_2_-X identifying a previously unknown mechanism of asthma in which the increase in TGM2 in the airways may function to amplify airway inflammation through eicosanoid generation.

Some of the results of this study have been previously reported in the form of abstracts [16], [17].

## Methods

Detailed methods are provided in the online supporting information (Text S1).

### Study Subjects

The University of Washington Institutional Review Board approved the study protocols, and written informed consent was obtained from all participants. Subjects 18–59 years of age were recruited who had a physician diagnosis of asthma for ≥1 year, and used only an inhaled β_2_-agonist for asthma treatment. In accordance with *a priori* definitions, asthmatics with a methacholine PC_20_≤4 mg/ml were identified with ≥20% fall in FEV_1_ following exercise challenge (EIB^+^ group) and asthmatic controls without EIB were identified with ≤5% fall in FEV_1_ following exercise challenge (EIB^−^ group). The first 3 subjects in each group were enrolled into the initial cohort, and the subsequent subjects were enrolled into the replication cohort.

Comparisons were also made to non-asthmatic subjects with negative methacholine (PC_20_>8 mg/ml) and dry air exercise challenge tests (<5% fall in FEV_1_ following exercise). Epithelial brushings and endobronchial biopsies were obtained from an additional group of subjects with asthma and EIB defined by a methacholine PC_20_≤4 mg/mL and ≥15% fall in FEV_1_ following exercise challenge.

### Study Protocol

Study subjects had spirometry and exercise challenge testing on one day, and a methacholine challenge on a separate day to determine eligibility for the study. The Seattle Asthma Severity and Control Questionnaire (SASCQ) assessed asthma control [18]. Eligible participants had 2 induced sputums conducted at baseline and one induced sputum 30 min after exercise challenge, each 2–10 days apart. Spirometry, exercise, and methacholine challenges were conducted in accordance with American Thoracic Society (ATS) standards [19], [20]. Induced sputum was conducted with 3% hypertonic saline via an ultrasonic nebulizer (DeVilbiss, Somerset, PA, USA) [1].

### RNA Isolation and Microarray Hybridization

The lower airway portion of the induced sputum was selected using a transfer pipette and dispersed in dithiothreitol 0.1% at 20°C. The cell pellet was immediately treated with caotropic lysis buffer and total RNA extracted. Biotin-labeled cRNA was prepared from 2–5 µg RNA from each sample, and after purification and fragmentation, hybridized with the Affymetrix human U133A array in the initial cohort and the U133 Plus 2 array in the replication cohort (Affymetrix, Santa Clara, CA). All the microarray data are MIAME compliant and have been deposited in GEO (Gene Expression Omnibus) under accession number GSE13785.

### Confirmation of Differentially Expressed Genes by Quantitative PCR, Western Blots, and Immunohistochemistry

Differences in RNA levels of selected differentially expressed genes were validated with qPCR using Taqman probes.[21] Western blots measured differences in the level of TGM2 in induced sputum supernatant and in epithelial lysates. TGM2 was localized in endobronchial tissue by the indirect immunoperoxidase technique using a rabbit polyclonal anti-TGM2 antibody.

### TGM2-mediated Activation of sPLA_2_ Function

The ability of TGM2 to increase the enzymatic activity of sPLA_2_s *in vitro* was tested by monitoring the release of free fatty acid from [^3^H]oleate-labeled *E. coli* membranes.[22] The measurements of sPLA_2_ activity were conducted initially using bovine pancreatic sPLA_2_ group 1B, and then recombinant human sPLA_2_-X [23]. Studies were conducted with purified TGM2 from guinea pig liver and then subsequently with rhTGM2. TGM2 was pre-incubated at concentrations ranging from 0.1 to 50×10^−3^ U/reaction with sPLA_2_s at 37°C for 15 min. Controls were conducted using TGM2 at a concentration of 10×10^−3^ U/reaction after the enzyme was heat denatured at 100°C for 10 min and with the active site of the enzyme saturated with N-carbobenzoxy-Gln-Gly.

### Statistical Analysis

The analytical approach was based on murine studies that compared asthma phenotype [24], [25] and on a human study comparing gene expression before and after allergen challenge [26]. The raw array data were normalized with GC Robust Multiarray Algorithm (GCRMA) [27]. Differential gene expression between the groups in the initial data set and the replication data set was determined with the linear models for microarray data (limma) package and P-values were calculated with a modified t-test in conjunction with an empirical Bayes method to moderate the standard errors of the estimated log-fold changes [28]. Two approaches were used to determine genes with differential expression between the groups, and between conditions (baseline and post-exercise). Genes with the most reproducible differential expression were identified by selecting genes in the initial and replication data sets with Log_2_FC≥1 and *P≤0.05*, and then assessing the combined statistical significance using the Fisher's combined P method [29]. A Bonferroni's correction assuming a correlation among genes of 0.6 was used to establish the P value cutoff. Based on the expression of 22,000 genes, a p value<0.05, a false discovery rate of 10%, and fold change of 2 (Log_2_ ratio of 1), with a standard deviation of 0.3, a sample size of 3 subjects per group gives 80% power in each the initial and replication cohorts [30]. The second approach was to determine the overall statistical significance of the combined data sets using Fisher's combined P method and the q-value to account for multiple testing based on a specified false discovery rate (FDR) [31]. Simple averaging combined the fold change values from each of the two platforms.

## Results

### Comparison of Asthma Phenotypes

**Figure 1:**
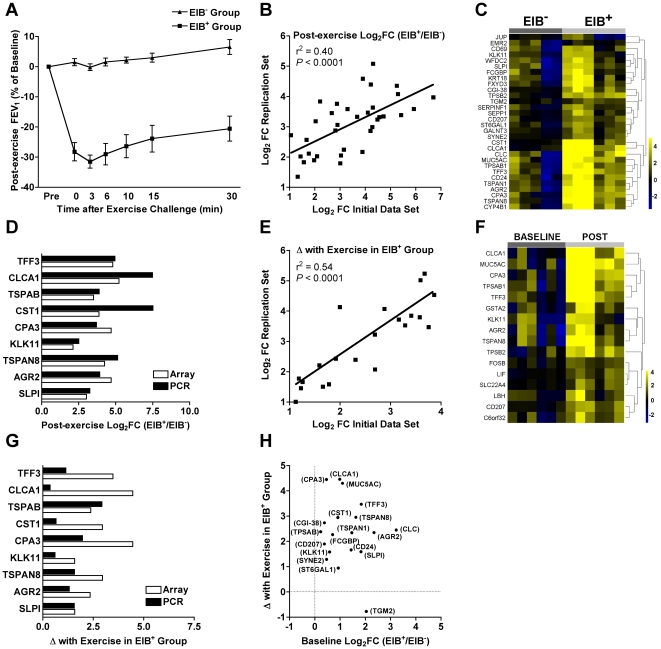
Comparison of lung function and gene expression between asthmatics with EIB and an asthmatic control group without EIB. The severity of EIB was markedly greater in the EIB^+^ group (**A**). Genes that were increased in the EIB^+^ group relative to the EIB^−^ group post-exercise were consistently differentially expressed in the initial and replication cohorts as shown by the strong association between differences in gene expression in each cohort (**B**). A heatmap of these genes shows the Log_2_FC relative to the average expression for each gene in each individual of the two groups post-exercise (**C**). The fold difference in gene expression between the groups post-exercise identified by the microarray platform was similarly demonstrated by qPCR (**D**). Within the EIB^+^ group, genes that increased in response to exercise challenge were consistently increased in the initial and replication cohorts (**E**). A heatmap of these genes shows the Log_2_FC relative to the average expression for each gene in each individual of the EIB^+^ cohort (**F**). The fold increase in gene expression in response to exercise challenge by the microarray platform was similarly demonstrated by qPCR with the exception of CLCA1 and KLK11 (**G**). To visualize the differences in the pattern of gene expression, differences in gene expression between the groups at baseline are plotted against the change in gene expression with exercise challenge in the EIB^+^ group (**H**).

We identified 15 subjects with asthma based on a methacholine PC_20_≤4 mg/ml, and enrolled 7 subjects who had EIB (≥20% fall in FEV_1_ post-exercise), and 7 subjects who did not have EIB (≤5% fall in FEV_1_ post-exercise) (Table 1). One additional subject was excluded due to an indeterminate response (>5% but <20% fall in FEV_1_ post-exercise) to exercise challenge. There were no differences in baseline lung function, asthma control, or response to the administration of a bronchodilator between the two groups. The reduction in FEV_1_ following exercise challenge was much greater in the EIB^+^ group as compared to the EIB^−^ group (*P*<0.001, Figure 1A) by definition. The EIB^+^ group had a slightly lower methacholine PC_20_ than the EIB^−^ group (*P* = 0.01), resulting in a modest relationship between the maximum fall in FEV_1_ after exercise challenge and the methacholine PC_20_ (r^2^ = 0.43, *P* = 0.01).

**Table 1:** Comparison of asthma phenotypes.*

	Asthma		
Characteristic	EIB^+^ (*n* = 7)	EIB^−^ (*n* = 7)	*P* value
Age (yr)			
Mean	27.7	32.7	0.33
Range	24–34	23–54	
Gender (% Male)	28.6	14.3	0.50
Race			
Caucasian	6	7	
Hispanic	1	0	
Baseline			
FEV_1_ (%)	88.0±9.4	85.9±9.6	0.68
FVC (%)	103.4±12.6	99.4±8.8	0.50
FEV_1_/FVC	0.72±0.06	0.73±0.11	0.84
FEF25–75 (%)	58.6±9.6	64.0±13.0	0.39
Post Bronchodilator			
Δ FEV_1_ (%)	7.1±3.5	8.9±5.7	0.51
Δ FVC (%)	−0.4±1.8	−0.0±1.72	0.69
Δ FEF25–75 (%)	22.8±7.1	20.1±7.1	0.49
Exercise-induced Bronchoconstriction			
Maximum Decrease in FEV_1_	−34.1±1.2	−1.2±2.0	<0.001
Area Under FEV_1_ Curve†	−749.7±288.4	95.7±75.7	<0.001
Direct Bronchial Hyperresponsiveness			
PC_20_ Methacholine‡	0.2 (0.1–0.4)	1.0 (0.4–2.5)	0.01
Asthma Control¥			
Nocturnal Symptoms	0.0±0.0	0.2±0.4	0.36
Daytime Symptoms	2.2±1.6	1.2±0.8	0.20
Activity Limitation	0.4±0.6	0.7±0.8	0.55
Asthma Exacerbations	1.0±1.4	0.7±1.2	0.68
Bronchodilator Use	1.6±2.1	1.3±2.0	0.83
SASCQ Summary Score	5.2±5.2	4.0±3.3	0.65
Asthma-free Days	19.2±9.7	24.2±4.6	0.29
Acute Asthma Visits	0.0±0.0	0.0±0.0	NS
Non-acute Asthma Visits	0.4±0.5	1.2±1.2	0.21

* Values reported are mean±standard deviation unless otherwise specified.

† Area under the FEV_1_ curve over the first 30 min after exercise (% change*min).

‡ Value represent the geometric mean (95% confidence interval). The *P* value is for the log-transformed value.

¥ Asthma control questions are from the Seattle Asthma Severity and Control Questionnaire (SASCQ) on a 0–5 point scale.

We isolated total RNA from leukocytes and epithelial cells in the lower airway portion of induced sputum that was selected and removed from salivary contamination.[32] The reproducibility of cellular constituents from induced sputum was very high (Figure S1 and Table S1). A comparison of induced sputum cellular constituents revealed minimal differences in the concentrations of leukocytes and epithelial cells between the groups (Figure S2 and Tables S2, S3, S4, S5). One subject from each group had insufficient RNA for the array analysis, but was included in the qPCR analysis. RNA quality was verified by capillary gel electrophoresis, and oligonucleotide microarrays were successfully hybridized for all 24 samples (6 EIB^+^ and 6 EIB^−^, baseline and post-exercise); however, one of the EIB^−^ post-exercise microarrays was excluded because of an unacceptably high normalized unscaled standard errors metric as well as failure to meet the manufacturer's quality control guidelines for housekeeping control probe signals which failed the threshold test.

### Differential Gene Expression Between Asthmatic Phenotypes

Gene expression in airway cells was analyzed between the two phenotypically distinct groups of asthmatics at baseline, and between the groups after exercise challenge. We used two methods to identify genes with the most reproducible differential expression between the groups. In the first method, we identified genes that were differentially expressed in the initial cohort (first 3 subjects in each group) and in the replication cohort (last three subjects in each group), and narrowed the list of genes to those with Log_2_ [fold change] (Log_2_FC)≥1.0 and *P*≤0.05 in both cohorts. The genes identified by this method represent genes with reproducible differential expression in both cohorts as demonstrated by the strong association between the magnitude of differential expression identified in each cohort (r^2^ = 0.40, *P*<0.0001, Figure 1B) and by differences between the two groups demonstrated by a heatmap showing the Log_2_FC relative to the average expression for each gene in each individual of the two groups post-exercise (Figure 1C). The combined statistical significance of these two sets was assessed, and a conservative Bonferroni correction was applied to identify 1 gene with differential expression between the groups at baseline and 19 genes with differential expression between the groups post-exercise (Table 2). We also assessed differences between the groups using the combined *P* value for the entire data set (Tables S6, S7). Based on a false discovery rate (FDR) of 10%, 28 genes in the post-exercise comparison were differentially expressed, including all 19 meeting the criteria for reproducible differential expression and 9 additional genes including CLCA2, GPR56, PROM1, TMC5, PFN2, IQCG, EFHC1, SERPINB2, and an expressed sequence tag (AV720803). Differences in the expression of 10 of these genes were confirmed by qPCR using samples from all individuals in each group, demonstrating a high degree of reproducibility by array or qPCR methods (Figure 1D).

**Table 2:** Genes with differential increased expression in the EIB^+^ group.

GenBank	Log_2_FC	*P* value	Symbol	Description
Baseline				
BC003551	2.04	0.0002	TGM2	Transglutaminase 2
Post-exercise				
NM_003226	4.80	0.0000	TFF3	Trefoil factor 3
AF127036	5.23	0.0000	CLCA1	Chloride channel, Ca^2+^-activated, member 1
NM_024164	3.50	0.0000	TPSB2	Tryptase β2
NM_001898	3.87	0.0000	CST1	Cystatin SN
NM_003890	2.85	0.0000	FCGBP	Fc fragment of IgG-binding protein
NM_001870	4.70	0.0000	CPA3	Carboxypeptidase A3 (mast cell)
AF206667	3.94	0.0000	TPSAB1	Tryptase α/β1
NM_006853	2.12	0.0000	KLK11	Kallikrein-related peptidase 11
NM_004616	4.25	0.0000	TSPAN8	Tetraspanin 8
AF088867	4.69	0.0000	AGR2	Anterior gradient homolog 2
AI743792	2.06	0.0000	ST6GAL1	ST6 β-galactosamide α-2,6-sialyltransferase 1
NM_003064	3.03	0.0001	SLPI	Secretory leukocyte peptidase inhibitor
AI521646	4.76	0.0002	MUC5AC	Mucin 5AC, oligomeric mucus/gel-forming
NM_016140	3.02	0.0002	CGI-38	Brain-specific protein
NM_015180	1.73	0.0002	SYNE2	Spectrin repeat-containing, nuclear envelope 2
NM_015717	1.93	0.0003	CD207	CD207, langerin
NM_001828	5.34	0.0003	CLC	Charcot-Leyden crystal protein
AK000168	4.03	0.0006	CD24	CD24
AF133425	3.97	0.0007	TSPAN1	Tetraspanin 1

### Genes Expression Response to Exercise Challenge

Because the bronchoconstrictor response to exercise challenge is a distinguishing feature of the EIB phenotype, the gene expression response to exercise challenge was assessed. We identified genes transcriptionally activated by exercise challenge in the initial cohort and replication cohorts of EIB^+^ subjects, and narrowed the list to genes with reproducible differential expression in both cohorts as demonstrated by the strong association of the differentially expressed genes in each cohort (r^2^ = 0.54, *P<0.0001*, Figure 1E), and by differences between the baseline and post-exercise conditions demonstrated by a heatmap showing the Log_2_FC relative to the average expression for each gene in each individual of the EIB^+^ cohort (Figure 1F). After applying a Bonferroni correction, 9 genes had increased expression and no genes had decreased expression following exercise challenge in the EIB^+^ group (Table 3). We also assessed changes in gene expression after exercise challenge using the combined *P* value for the EIB^+^ phenotype (Table S8). Based on a FDR of 10%, 8 genes had increased expression following exercise challenge in the EIB^+^ group, including 3 additional genes CLCA2, FCGBP, and CST1 not observed in our initial selection algorithm. We confirmed exercise-induced increases in expression of 7 of these genes (TFF3, TPSAB, CPA3, KLK11, TSPAN8, AGR2, and SLPI) by qPCR (Figure 1G). In the EIB^−^ group, no gene met either criterion for change in expression in response to exercise challenge.

**Table 3:** Genes in EIB^+^ group with increased expression in response to exercise challenge.

GenBank	Log_2_FC	*P* value	Symbol	Description
NM_024164	3.69	0.0000	TPSB2	Tryptase β2
AF127036	4.46	0.0000	CLCA1	Chloride channel, Ca^2+^-activated, member 1
NM_003226	3.48	0.0000	TFF3	Trefoil factor 3 (intestinal)
NM_003294	3.63	0.0000	TPSAB1	Tryptase α/β1
NM_001870	4.46	0.0000	CPA3	Carboxypeptidase A3 (mast cell)
NM_006853	1.58	0.0002	KLK11	Kallikrein-related peptidase 11
NM_015717	1.90	0.0003	CD207	CD207 molecule, langerin
AI521646	4.31	0.0004	MUC5AC	Mucin 5AC, oligomeric mucus/gel-forming
NM_004616	2.96	0.0007	TSPAN8	Tetraspanin 8

We also evaluated in the change in gene expression in response to challenge in the EIB^+^ group relative to the change in expression in the EIB^−^ group (i.e. interaction effect, Table S9) and found that 7 genes TPSB2, CLCA1, FCGBP, CST1, TPSAB1, CPA3, and TFF3 demonstrated increased relative expression based on the reproducibility criteria, and an additional gene CLCA2 based on the FDR.

To visualize the differences in the pattern of gene expression, differences in gene expression between the groups at baseline are plotted against the change in gene expression with exercise challenge in the EIB^+^ group (Figure 1H). Only transglutaminase 2 (TGM2) was differentially expressed at baseline, while other genes including the mast cell proteases were not differentially expressed at baseline, but had a marked increase following exercise challenge. Other genes such as trefoil factor 3 (TFF3) had modest increase in expression at baseline and further increase after exercise challenge.

### Confirmation of Differentially Expressed Genes in the Airways

**Figure 2:**
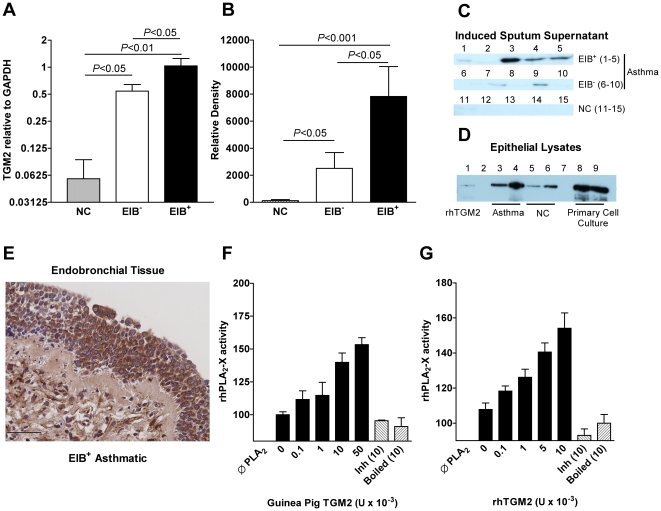
Comparison of TGM2 levels in the airways and *in vitro* function of TGM2 in the activation of secreted PLA_2_ activity. The gene expression of TGM2 by qPCR in airway cells was increased in both asthma groups relative to non-asthmatic controls, and the expression was higher in the EIB^+^ group (**A**). TGM2 was not detected in induced sputum supernatant from non-asthmatic controls, but was elevated in asthmatics, especially in the EIB^+^ group (**B**). The bands from one of two Western blots used to quantify the levels of TGM2 in induced sputum supernatant are shown in the panel, with lanes 1–5 representing EIB^+^ asthmatics, lanes 6–10 representing EIB^−^ asthmatics, and lanes 11–15 normal controls (**C**). A Western blot of epithelial brushings from asthmatics demonstrates higher levels in the epithelium from asthmatics relative to non-asthmatic controls (**D**). In the blot, the first lane is recombinant human TGM2 (rhTGM2), lanes 3 and 4 are epithelial lysates from 2 different EIB^+^ asthmatics, and lanes 5 and 6 are epithelial lysates from 2 different normal controls. Asthmatic epithelial cells from an EIB^+^ asthmatic in primary culture shown in lanes 8 and 9 also strongly express TGM2. Immunostaining for TGM2 in airway biopsies from an EIB^+^ asthmatic demonstrates immunostaining in the airway epithelium for TGM2 (40×, scale bar is 50 µm) (**E**). Pre-incubation of recombinant human sPLA_2_-X with purified TGM2 from guinea pig liver (**F**) or with recombinant human TGM2 (**G**) causes an increase in the PLA_2_ activity of the sPLA_2_-X enzyme. Denaturing the TGM2 with heat (boiled) or inhibiting the activity of the enzyme by saturating the enzyme with N-carbobenzoxy-Gln-Gly (Inh) demonstrate that the *in vitro* findings are due to the enzymatic activity of TGM2.

We focused on TGM2 because it was the only gene that was differentially expressed between the groups at baseline. Because the difference in TGM2 expression between the two asthma groups was modest in magnitude, we also assessed the expression of TGM2 by qPCR in a non-asthmatic control group demonstrating that the expression of TGM2 was increased in both asthma groups relative to the non-asthmatic group, and that it was further increased in asthmatics with the EIB^+^ phenotype (Figure 2A). The 85 kDa TGM2 protein was essentially undetectable from the non-asthmatic group, but measurable in most of the asthmatic samples, and increased in the EIB^+^ asthmatic group (Figures 2B–C). The oligonucleotide probe and qPCR primer measure a portion of the gene that is common to both the full-length gene and the recently described splice variant [33]. In epithelial brushings from asthmatics with EIB, the level of TGM2 was increased relative to non-asthmatic controls, and the level of TGM2 was markedly increased in airway epithelial cells in primary culture from an EIB^+^ asthmatic individual (Figure 2D). Immunohistochemistry of airway biopsies taken from conducting airways of 3 EIB^+^ asthmatic individuals demonstrated strong immunostaining for TGM2 in the epithelial layer (Figure 2E and Figure S3)

### Effects of Transglutaminase 2 on Native and Recombinant sPLA_2_ Activity

The ability of TGM2 to increase the enzymatic activity of sPLA_2_-X *in vitro* was tested by monitoring the release of free fatty acid from [^3^H]oleate-labeled *E. coli* membranes [22]. Recombinant human sPLA_2_-X was generated in *E. coli*, purified and refolded to the active enzyme [23]. The initial experiments were conducted with purified TGM2 from guinea pig liver, and then subsequently with recombinant human TGM2. Pre-incubation of sPLA_2_-X at 37°C for 15 min with guinea pig TGM2 caused a concentration dependent increase in the enzyme activity of sPLA_2_-X (Figure 2F). Similarly, the human recombinant TGM2 caused a concentration-dependent increase in sPLA_2_-X activity (Figure 2G). To further demonstrate that the increase in sPLA_2_-X was due to the enzymatic activity of the TGM2, we found that heat denaturing the TGM2 enzyme prior to the experiment, or saturating the active site of the enzyme with N-carbobenzoxy-Gln-Gly inhibited the activation of sPLA_2_-X (Figures 2F–G).

## Discussion

Relatively little is known about the immunological basis of indirect AHR, an aspect of asthma that is manifested by the presence of airway narrowing triggered by exercise, hypertonic aerosols, cold air and adenosine [7]. In a recent large longitudinal study of children, cold-air AHR was one of the strongest predictors of persistent asthma in adulthood [34]. In the present study, we enrolled subjects with a rigorous diagnosis of asthma, and identified subjects with and without EIB, a syndrome characterized by an abnormal airway epithelium and increased concentrations of inflammatory eicosanoids in the airways [8]. Through analysis of airway gene expression before and after exercise challenge in these distinct groups, we found that genes related to epithelial repair and mast cell infiltration are increased in asthmatics with EIB, and that TGM2 is the most differentially expressed between the groups at baseline. We confirmed that the TGM2 protein is increased in airway cells and airway lining fluid in the EIB positive group, and that both asthma groups have increased levels relative to a non-asthmatic control group. We also found that the TGM2 protein is avidly expressed in the epithelium of the EIB positive asthmatics. Further, TGM2 causes a sustained increase in sPLA_2_-X activity, identifying a novel mechanism by which increased expression of TGM2 may serve to amplify airway inflammation in asthma.

The present study adds to an accumulating body of evidence from gene-array studies that genes such as CLCA1 [35], [36], SerpineB2 [26], [36], [37], MUC5AC [35], [37], AGR2 [35], CPA3 [36], [37], and tryptase [36], [37] are overexpressed in asthma, and suggests that these genes are more transcriptionally active in the EIB phenotype. A further advance in the present study was the evaluation of gene expression in response to exercise challenge that induces acute asthma in susceptible subjects demonstrating that several of the genes are transcriptionally activated during acute asthma. We chose an early time point following challenge in this study to minimize secondary transcriptional activation in response to the release of mediators in the airways. Several common gene expression programs emerge with increased expression in the EIB phenotype notable for genes related to epithelial repair and differentiation including TFF3, TGM2, TSPN8, TSPN1, SLPI, and CD24, as well as regulation of epithelial lining fluid and mucin production including CLCA1, MUC5AC, and AGR2. In addition, proteases including the mast cells proteases TPSB2 and CPA3 as well as KLK11 and protease inhibitors such CST1 and SLPI are notably increased in the EIB positive group. These findings provide new insights into the pathogenesis of acute asthma, and suggest that genes related to epithelial repair are more active in asthma, and are particularly active in the EIB phenotype.

Increased transcription of mast cell proteases tryptase and CPA3 in the EIB^+^ group following exercise challenge adds additional evidence that mast cell activation plays an important role in the pathophysiology of EIB. We have previously demonstrated mast cell activation following exercise challenge in EIB as evidenced by release of mast cell-specific mediators histamine and tryptase [1]. A notable finding in the present study is that CPA3, a secretory granule metalloexopeptidase that is predominantly expressed in mast cells of the MC_TC_ type was increased along with tryptase. Mast cells are functionally divided into MC_T_ and MC_TC_ types based on the composition of their secretory granules that contain tryptase in both types of cells, but with the addition of chymase, carboxypeptidase A3, and cathepsin G in the MC_TC_ type. Increased expression of both tryptase and CPA3 was also recently noted in the airway epithelium in asthma [36], suggesting that the expression of these proteases are increased in asthma, and further increased in patients with EIB.

We found that TGM2 gene expression and secreted protein are increased in the airways of subjects with asthma, and further increased in the EIB^+^ phenotype. Although TGM2 has been implicated in a number of inflammatory diseases, and has been shown to be upregulated by retinoic acid in transformed airway epithelial cells [38], it has not been previously implicated in asthma. The TGM2 gene is located on chromosome 20q11.2-12 near a cluster of genes related to epithelial barrier function in close proximity to a region linked to both atopic dermatitis and asthma [39]. Two of the other differentially expressed genes in the present study, SLPI and CST1, are also located in this region of chromosome 20. TGM2 is a calcium-dependent enzyme that modifies protein structure through the transfer of an acyl group from glutamine to lysine or free amines resulting in a new inter- or intra-molecular amidic cross-link that may directly alter the enzyme activity of sPLA_2_ [40]. In studying the function of TGM2 in asthma, we demonstrated that TGM2 enzymatically modifies sPLA_2_-X leading to a substantial increase in the PLA_2_ activity of the enzyme. This finding has strong implications for the pathophysiology of asthma since PLA_2_ catalyzes the first rate-limiting step leading to eicosanoids such as CysLTs that play an important role in asthma. Our prior research has found that EIB^+^ asthmatics have increased levels of CysLTs and CysLT/PGE_2_ ratio relative to asthmatics without EIB [8] and elevated levels of 15*S*HETE relative to non-asthmatic controls [14]. Although there are 9 functional human sPLA_2_s [41], we focused on the functional alteration of sPLA_2_-X because we have recently found that sPLA_2_-X is increased in the airways of asthmatics, and may be involved in eicosanoid generation in the airways post-exercise challenge [14]. Further, deletion of the sPLA_2_-X gene in a murine model of asthma markedly inhibits the development of airway inflammation, AHR, Th2 cytokine production, and structural remodeling [15]. In prior investigations, TMG2 from guinea pig liver has been shown to increase the PLA_2_ activity of bovine pancreatic PLA_2_, and dual inhibitors of TGM2 and sPLA_2_ reduced ocular inflammation in a rabbit model of allergen-induced conjunctivitis [40]. In addition to regulating the release of free arachidonate, sPLA_2_s are also involved in the generation of lysophospholipids and the degradation of surfactant phospholipids implicated in asthma pathogenesis [41]. In addition to effects on eicosanoid metabolism TGM2 also activates the transcription factor NFκB that is broadly implicated in the generation of pro-inflammatory cytokines in asthma and other inflammatory diseases, and induces iNOS via NFκB [42].

In summary, we have identified novel mediators of asthma using genome-wide expression profiling in phenotypically distinct groups of asthmatics. We found that processes related to epithelial repair and mast cell infiltration were increased in the asthma group that had EIB. Functional analyses demonstrated TGM2 causes a sustained increase in sPLA_2_-X activity. Because PLA_2_ is an enzyme that catalyses the first rate-limiting step leading to eicosanoids, and specifically sPLA_2_-X has recently been identified as a potential mediator of AHR in a murine model of asthma and in human subjects with asthma, these findings may reveal a novel mechanism that functionally serves to amplify airway inflammation through the generation of inflammatory eicosanoids known to be increased in the EIB phenotype.

## Supporting Information

Text S1 (0.12 MB DOC)

Figure S1 Bland-Altman plots for the reproducibility of the concentration of leukocytes and epithelial cells in induced sputum. The plots represent the concentration of lower airway cells (A), and percentage of eosinophils (B), lymphocytes (C), macrophages (D), neutrophils (E), and columnar epithelial cells (F) in induced sputum. The Bland-Altman plot summarizes the difference in the measure between two visits versus the average value of the measure for the two visits for each subject. Each plot shows the overall average difference (solid line)±2 standard deviations (dashed line). These results demonstrate that the induced sputum cell counts are reproducible because the individual points on the Bland-Altman plot are randomly scattered around the overall average difference, and most points fall within 2 standard deviations of the overall average difference.
(0.36 MB TIF)

Figure S2 Comparison of the concentrations of inflammatory and epithelial cells in induced sputum before and after exercise challenge. The baseline and post-exercise induced sputum tests were conducted on average 6.5 days apart. There were no significant changes in the concentrations of eosinophils (Eos), lymphocytes (Lymph), macrophages (Mac), and neutrophils (PMN) in induced sputum in EIB+ asthmatics (A) or a group of EIB- asthmatic controls (B). The concentration of epithelial cells (Epi) increased significantly in the EIB+ group, and a similar trend occurred in the EIB- control group.
(0.58 MB TIF)

Figure S3 Immunostaining for TGM2 in biopsies of the airway epithelium of EIB+ asthmatics. Immunostaining for TGM2 is strong in the airway epithelium and surrounding matrix of endobronchial biopsy from an EIB+ asthmatic (A). Non-specific immunostaining was not observed using an isotype control antibody (B).
(3.20 MB TIF)

Table S1 Reproducibility of cellular components of induced sputum on 2 separate visits
(0.03 MB DOC)

Table S2 Baseline differences in selected induced sputum*
(0.05 MB DOC)

Table S3 Post-exercise Differences in Selected Induced Sputum*
(0.05 MB DOC)

Table S4 Regression analysis of differences in selected induced sputum at baseline*
(0.04 MB DOC)

Table S5 Regression analysis of differences in selected induced sputum post-exercise*
(0.04 MB DOC)

Table S6 Genes with increased expression in EIB+ group relative to EIB- group at baseline (Log_2_FC>1, *P<0.05*)
(0.04 MB DOC)

Table S7 Genes with differential expression in EIB+ group relative to EIB- group post exercise (Log_2_FC>1, *P<0.05*)
(0.13 MB DOC)

Table S8 Genes with change in expression after exercise challenge in the EIB+ group (Log_2_FC>1, *P<0.05*)
(0.08 MB DOC)

Table S9 Genes with changes in expression after exercise challenge (post-exercise minus baseline) in the EIB+ group relative to EIB- group (Log_2_FC>1, *P<0.05*)
(0.06 MB DOC)
