# Supplementary material for: Transglutaminase 2, a Novel Regulator of Eicosanoid Production in Asthma Revealed by Genome-Wide Expression Profiling of Distinct Asthma Phenotypes
Source: PLoS One. 2010 Jan 5;5(1):e8583. doi: 10.1371/journal.pone.0008583 (PMC2797392; doi:10.1371/journal.pone.0008583)
Supplement: Text S1 — (0.12 MB DOC) [file pone.0008583.s001.doc]

**Supplemental Material**

**Transglutaminase 2, a novel regulator of eicosanoid production in asthma revealed by genome-wide expression profiling of distinct asthma phenotypes**

Teal S. Hallstrand, Mark M. Wurfel, Ying Lai, Zhanglin Ni, Michael H. Gelb, William A. Altemeier, Richard P. Beyer, Moira L. Aitken, and William R. Henderson, Jr.

**Methods**

Study Subjects

The University of Washington Institutional Review Board approved the study protocols, and written informed consent was obtained from all participants. Subjects 18-59 years of age were recruited who had a physician diagnosis of asthma for ≥ 1 year, and used only an inhaled β_2_-agonist for asthma treatment. In accordance with *a priori* definitions, asthmatics with a methacholine PC_20_ ≤ 4 mg/ml were identified with ≥ 20% fall in FEV_1_ following exercise challenge (EIB^+^ group) and asthmatic controls without EIB were identified with ≤ 5% fall in FEV_1_ following exercise challenge (EIB^-^ group). The first 3 subjects in each group were enrolled into the initial cohort, and the subsequent subjects were enrolled into the replication cohort. Exclusion criteria included baseline FEV_1_ ≤ 65% predicted, history of smoking cigarettes within the prior year or ≥ 7 pack-year smoking, treatment for acute asthma within the prior month, hospitalization for asthma within the prior 3 months, or history of life-threatening asthma. Participants were excluded if they had used an inhaled or oral corticosteroid, leukotriene modifier, long-acting antihistamine, cromone, or long-acting β_2_-agonist in the 30 days prior to the study.

Comparisons were also made to non-asthmatic subjects with negative methacholine (PC_20_ > 8 mg/ml) and dry air exercise challenge tests (< 5% fall in FEV_1_ following exercise). The non-asthmatic control group samples were collected for a previously conducted study [1].

Epithelial brushings and endobronchial biopsies were obtained from subjects with asthma and EIB defined by a methacholine PC_20_ ≤ 4 mg/mL and ≥ 15% fall in FEV_1_ following exercise challenge. Some epithelial brushings from EIB^+^ asthmatics were placed in primary culture and used at passage 1 or 2 for *in vitro* experiments.

Study Protocol

The first visit consisted of a physical examination, spirometry, and exercise challenge to determine eligibility for the study. Participants with > 5%, but < 20% decline in FEV_1_ after exercise challenge were excluded from the study. The Seattle Asthma Severity and Control Questionnaire (SASCQ) assessed asthma control [2]. On the second visit, all participants had a methacholine challenge. Participants with a methacholine PC_20_ > 4 mg/ml were excluded. The third through fifth visits were conducted at the same time of day, 2-10 days apart. On visits three and four, all participants had spirometry before and 15 min after administration of 180 μg of albuterol via a metered dose inhaler, followed by induced sputum. On the fifth visit, all participants had induced sputum conducted 30 min after the conclusion of exercise challenge. Fifteen min after exercise, 180 μg of albuterol via a metered dose inhaler was administered to all subjects. Subjects were asked not to exercise, use short-acting antihistamines for 48 hours, or β_2_-agonist or caffeinated beverages for 6 hours prior to each study visit.

Spirometry, Exercise, and Methacholine Challenge

Spirometry, exercise, and methacholine challenges were conducted in accordance with American Thoracic Society (ATS) standards [3]. Exercise challenge was performed on a motorized treadmill such that each subject sustained ≥ 85% of their maximum heart rate for the final 6 min of exercise [4]. Subjects wore nose clips and breathed dry air (0% RH, 22^o^C) delivered from a weather balloon reservoir through a one-way valve (Hans Rudolph, Kansas City, MO) during exercise. Spirometry was conducted 20 and 5 min before each exercise challenge, and repeated at 0, 3, 6, 10, 15, and 30 min after the end of exercise. The better of at least 2 FEV_1_ maneuvers within 5% of each other was recorded at each time point. Methacholine challenge was conducted using a dosimeter [4].

Sputum Induction

Induced sputum was conducted with 3% hypertonic saline via an ultrasonic nebulizer (DeVilbiss, Somerset, PA, USA) [5]. At 2-min intervals, subjects were asked to clear saliva from their mouth and then expectorate sputum. Sputum was collected over 20 min and was pooled into a single sample container. The induced sputum was placed on ice immediately and processed within 15 min of collection. Samples were coded with a subject number, visit number, and date. The link between the clinical characteristics of the participants and the coded labels were maintained in a separate file by the principal investigator. A laboratory technician who was blinded to the clinical characteristics of each participant performed total and differential cell counts.

RNA Isolation and Microarray Hybridization

The lower airway portion of the induced sputum was selected using a transfer pipette and dispersed in dithiothreitol 0.1% (Calbiochem, La Jolla, CA) in a cool shaking water bath at 20^o^C for 15 min. A portion of the homogenized sputum was used to assess cell viability, cell count and cell differential. The dispersed induced sputum sample was centrifuged at 250 *g* for 10 min, the supernatant removed, and the cell pellet was immediately treated with caotropic lysis buffer. Total RNA was extracted using the RNeasy Mini protocol (Qiagen, Valencia, CA). The integrity of RNA was assessed with an Agilent Bioanalyzer (Agilent, Santa Clara, CA). Biotin-labeled cRNA was prepared from 2-5 µg RNA from each sample, and after purification and fragmentation, hybridized with the Affymetrix human U133A array in the initial cohort and the U133 Plus 2 array in the replication cohort (Affymetrix, Santa Clara, CA). After hybridization, each microarray was washed, stained, and amplified on the Affymetrix Fluidics Station and the images read using the GeneChip Scanner 3000 (Affymetrix). All the microarray data are MIAME compliant and have been deposited in GEO (Gene Expression Omnibus) under accession number GSE13785.

Research Bronchoscopy

Research bronchoscopy was conducted with an additional group of subjects with asthma and EIB defined by a methacholine PC_20_ ≤ 4 mg/ml and ≥ 15% fall in FEV_1_ following exercise challenge. Research bronchoscopy was also conducted with a group of non-asthmatic controls with a methacholine PC_20_ > 8 mg/ml and < 7.5 % fall in FEV_1_ following exercise challenge. Bronchoscopy was conducted on a separate day without exercise provocation. During bronchoscopy, 4 epithelial brushings were obtained from 2^nd^ to 5^th^ generation airways of the left lower lobe and lingual. Mucosal biopsies were obtained from 2^nd^ to 5^th^ generation carina of the right lower and middle lobes using a 1.8 mm forceps.

Quantitative Real-time PCR

Differences in RNA levels of differentially expressed genes were validated with qPCR using Taqman probes as described previously.[6] A 230 ng aliquot of RNA was used for first strand cDNA synthesis using oligo (dT) primers (SuperScript, Invitrogen, Carlsbad, CA). Primer-probe sets for selected genes were obtained from the Applied Biosystems Assays-on-Demand repository (Applied Biosystems, Foster City, CA). The reference numbers are as follows: AGR2, Hs00180702_m1; CLCA1, Hs00154490_m1; CPA3, Hs00157019_m1; CST1, Hs00606961_m1; KLK11, Hs01100849_m1; SLPI, Hs00268204_m1; TFF3, Hs00173625_m1; TGM2, Hs00190278_m1; TSPAB, Hs02576518_gH; TSPAN8, Hs00610327_m1; and GAPDH, Hs99999905_m1. Real-time PCR was performed using an ABI PRISM 7900 (Applied Biosystems) with the following conditions: 95°C for 15 sec, 60°C for 1 min repeated for 40 cycles. Quantities of the specific transcripts were determined by comparing Ct values observed in each sample with Ct values obtained from a dilution series of reverse transcribed pooled reference RNA. Values obtained for each transcript of interest were normalized to the level of GAPDH mRNA detected in each sample.

Western Blots

Western blots were conducted to measure differences in the levels of TGM2 in induced sputum supernatant and in epithelial lysates. The protein concentration was measured with the Coomassie Plus Bradford assay (Pierce, Rockford, IL). 5 randomly selected induced sputum supernatant samples from each of the asthma groups and 5 normal controls were prepared under reducing conditions with heating to 100^o^C for 10 min. 5 μg of total protein was loaded from each sample and separated on a 4-12% NuPage Bis-Tris gel (Invitrogen) at 200V for 28 min. Proteins were transferred by semi-dry transfer to PVDF membranes at 110 mAmp for 40 min. Non-specific binding was blocked with 5% nonfat milk in TBST for 2 hours at RT. The membrane was incubated with rabbit polyclonal anti-TGM2 antibody overnight at 4^o^C (Santa Cruz Biotechnology, Santa Cruz, CA), and then subsequently incubated with goat anti-rabbit HRP-linked antibody (Cell Signaling, Danvers, MA) for 1 hour at RT. The peroxidase activity was detected using LumiGLO ECL reagents (Cell Signaling, Danvers, MA). Initial studies demonstrated that the control protein, recombinant human TGM2, ran in the same location as the band from induced sputum supernatant, and subsequently the control protein was omitted so that 5 samples from each group could be run simultaneously. Two Western blots were conducted each containing 5 randomly selected samples from each group and the results combined in the final analysis. Similarly, 5 μg of total protein from epithelial lysates from brushings of the lower airways from asthmatics with EIB, non-asthmatic subjects, and asthmatic epithelial cells in primary culture were measured by Western blot.

Immunohistochemistry

Endobronchial biopsies were fixed in methyl Carnoy's and embedded in paraffin. A rabbit polyclonal anti-TGM2 antibody (Santa Cruz Biotechnology) was used to localize the TGM2 in endobronchial tissue by the indirect immunoperoxidase technique. Endogenous peroxidase activity was blocked with 3% hydrogen peroxide, and endogenous biotin activity was blocked with the Avidin-Biotin blocking Kit (Vector Laboratories, Burlingame, CA). The primary antibody diluted in phosphate-buffered saline containing 1% BSA was followed by the biotinylated secondary antibody (Vector Laboratories), and the avidin-biotin-horseradish peroxidase (HRP) complex (Vector Laboratories). The immunoreaction was visualized by 3,3'-diaminobenzidine (DAB; Sigma-Aldrich) with nickel chloride enhancement, resulting in a brown-black color product.

**Primary Respiratory Epithelial Cell Culture**

Primary bronchial epithelial cells were isolated from EIB^+^ asthmatics during bronchoscopy using a nylon cytology brush of cells from subsegmental airways. Epithelial cells from the cytology brush were suspended in bronchial epithelial basal media (BEBM, Lonza, Allendale, NJ) supplemented with bovine pituitary extract, insulin, hydrocortisone, gentamicin, amphotericin B, fluconazole, retinoic acid, transferrin, triiodothyronine, epinephrine, and human recombinant epidermal growth factor (serum-free BEGM). The cells were then seeded into a culture vessel (25 cm^2^ growth surface area) pre-coated with type 1 collagen and maintained at 37°C in a humidified incubator. Cells were cryopreserved and/or subcultured at 90-95% confluence. Primary cultures of cryopreserved asthmatic epithelial cells from passage 1 were used to measure the TGM2 protein in cultured cells by Western blot.

TGM2-mediated Activation of sPLA_2_ Function

The ability of TGM2 to increase the enzymatic activity of sPLA_2_s *in vitro* was tested by monitoring the release of free fatty acid from [^3^H]oleate-labeled *E. coli* membranes [7]. The measurements of sPLA_2_ activity were conducted initially using bovine pancreatic sPLA_2_ group 1B (sPLA_2_-1B, EC 3.1.1.4, Sigma-Aldrich), and then human sPLA_2_-X. Recombinant human sPLA_2_-X was generated in *E. coli*, purified and refolded to the active enzyme [8]. The initial experiments were conducted with purified TGM2 from guinea pig liver (EC 2.3.2.13, Sigma-Aldrich) and then subsequently with rhTGM2 (Zedira). The activity of the TGM2 was confirmed by monitoring the kinetics of TGM2-mediated formation of hydroxamate from hydroxylamine and N-carbobenzoxy-Gln-Gly [9]. Guinea pig TGM2 was pre-incubated at concentrations ranging from 0.1 to 50 x 10^-3^ U/reaction with sPLA_2_-1B in 50 μl assay buffer (100 mM TrisHCl, pH 8.0, 10 mM CaCl_2_, 0.1% fatty acid free BSA) at 37^o^C for 15 min. After pre-incubation, ~200,000 dpm of [^3^H]oleate-labeled *E. coli* membranes in 100 μl of reaction buffer were added and the mixture incubated at 37^o^C for 1 hour. The addition of 300 μl of 0.1 M EDTA, pH 8.0, and 1% fatty acid-free BSA stopped the reaction. The samples were centrifuged at 13,000g for 3 min, and 400 μl of the supernatant was used for scintillation counting. Control incubations in the absence of sPLA_2_ were carried out in parallel to calculate specific hydrolysis. Additional controls were conducted using TGM2 at a concentration of 10 x 10^-3^ U/reaction after the enzyme was heat denatured at 100^o^C for 10 min and with the active site of the enzyme saturated with N-carbobenzoxy-Gln-Gly. Guinea pig TGM2 was then pre-incubated at concentrations ranging from 0.1 to 50 x 10^-3^ U/reaction with 25 pg of human recombinant sPLA_2_-X. Human recombinant TGM2 was then pre-incubated at concentrations ranging from 0.1 to 10 x 10^-3^ U/reaction with 25 pg of human recombinant sPLA_2_-X.

Statistical Analysis

The characteristics of the study participants were compared with unpaired *t*-tests for continuous variables and Chi square tests for categorical variables. The area under the FEV_1_/time curve (AUC)[10] quantified the severity of EIB over a 30-min period after exercise (AUC_30_). The relationship between methacholine PC_20_, and the severity of EIB was assessed with the Pearson’s correlation coefficient. The medians of differential cell counts were compared between different groups with the Mann-Whitney U test. Regression analysis was also conducted to determine differences in cell counts between the groups after adjustment for methacholine PC_20_. Reproducibility of induced sputum cell counts between the two baseline induced sputum samples was assessed by the concordance correlation coefficient, and by Bland-Altman plots [11]. Comparisons of the log transformed qPCR data were compared with an unpaired *t*-test or ANOVA with Newman-Keul’s post-hoc tests. Comparisons of the levels of proteins in induced sputum were made with a Mann-Whitney U test or a Kruskal-Wallis test with Dunn’s multiple comparison post-hoc tests.

The raw array data were normalized with GC Robust Multiarray Algorithm (GCRMA) using Bioconductor software ([www.biocondutor.org](http://www.biocondutor.org/)) [12]. Differential gene expression between the groups in the initial data set and the replication data set was determined with the linear models for microarray data (limma) package and *P*-values were calculated with a modified *t*-test in conjunction with an empirical Bayes method to moderate the standard errors of the estimated log-fold changes [13]. Two approaches were used to determine genes with differential expression between the groups, and between conditions (baseline and post-exercise). Genes with the most reproducible differential expression were identified by selecting genes in the initial and replication data sets with Log_2_FC ≥ 1 and *P* ≤ 0.05, and then assessing the combined statistical significance using the Fisher's combined *P* method [14]. A Bonferroni’s correction assuming a correlation among genes of 0.6 was used to establish the *P* value cutoff. The second approach was to determine the overall statistical significance of the combined data sets using Fisher's combined *P* method and the q-value to account for multiple testing based on a specified false discovery rate (FDR) [15]. Simple averaging combined the fold change values from each of the two platforms.

**Supplemental Results**

Comparisons of Asthma Phenotypes and Airway Hyperresponsiveness

We identified 15 subjects with asthma based on a methacholine PC_20_ ≤ 4 mg/ml, and enrolled 7 subjects who had EIB (≥ 20% fall in FEV_1_ post-exercise), and 7 subjects who did not have EIB (≤ 5% fall in FEV_1_ post-exercise) (Manuscript Table 1). One additional subject was excluded due to an indeterminate response (> 5% but < 20% fall in FEV_1_ post-exercise) to exercise challenge. There were no differences in baseline lung function or response to the administration of a bronchodilator between the two groups. There were no differences between the groups in the frequency of asthma symptoms, short-acting bronchodilator use, asthma-free days, and doctor visits for asthma. The reduction in FEV_1_ following exercise challenge was much greater in the EIB^+^ group as compared to the EIB^-^ group (*P* < 0.001, Manuscript Fig 1A). The EIB^+^ group had a slightly lower methacholine PC_20_ than the EIB^-^ group (*P* = 0.05), resulting in a relationship between the maximum fall in FEV_1_ after exercise challenge and the methacholine PC_20_ (r^2^ = 0.43, *P* = 0.01). However, there was no relationship between the severity of EIB measured by either the maximum fall in FEV_1_ or the AUC_30_ and the methacholine PC_20_ in the EIB positive group (r^2^ = 0.07, *P* = 0.58). There was no relationship between the severity of EIB measured by the maximum fall in FEV_1_ and baseline lung function (r^2^ = 0.09, *P* = 0.29) or response to a short acting-bronchodilator (r^2^ = 0.04, *P* = 0.50).

Comparison of Lower Airway Cellular Constituents

In this study, total RNA was isolated from the cellular component of induced sputum in which the lower airway portion was selected and removed from salivary contamination [16]. The reproducibility of cellular constituents from induced sputum was very high (Table S1, Figure S1). The concentrations of leukocytes and epithelial cells in the induced sputum at baseline were similar between the groups with the exception of the percentage, but not the concentration of eosinophils that was higher in EIB^+^ group relative to the EIB^-^ group (Table S2). The concentration of columnar epithelial cells from the lower airways was similar between the groups; this result differs from prior results showing a higher concentration in epithelial cells in unselected induced sputum in the EIB^+^ asthmatics relative to EIB^-^ asthmatics [17]. We attribute these differences to the sputum selection process, because the unselected portion of induced sputum that is removed contains some of the columnar epithelial cells (data not shown). In the post-exercise induced sputum, the percentage and concentration of eosinophils was higher in the EIB^+^ group relative to the EIB^-^ negative group, but there were no differences in the percentage or concentration of any of the other cellular constituents (Table S3). Because direct AHR to methacholine was greater in the EIB^+^ group in addition to the marked differences in severity of EIB, a regression analysis was used to adjust for differences in methacholine PC_20_ between the groups. Comparison of differences in cellular constituents in induced sputum was not appreciably altered after taking account of the difference in methacholine AHR between the groups using the regression analysis (Tables S4 and E5). There were no significant changes in the concentrations of leukocytes in the airways between baseline and post-exercise in either of the groups; however, the concentration of columnar epithelial cells increased following exercise in the EIB^+^ group, and a similar trend was observed in the EIB^-^ group (Figure S2).

The amount of total RNA isolated from induced sputum cells ranged from 0.7 μg to 10.9 μg, with an average of 3.5 μg (SD 2.4). Because we used at least 2 μg from each sample for the oligonucleotide arrays, one subject from each group had insufficient RNA for the array analysis, but was included in the qPCR analysis. The capillary gel electrophoresis demonstrated good RNA quality on all samples with the average rRNA ratio of 1.2. Arrays were successfully hybridized for all 24 samples (6 EIB^+^ and 6 EIB^-^, baseline and post-exercise). The 3'/5' ratio for glyceraldehyde-3-phosphate dehydrogenase (GAPDH) was 1.56 (SD 0.31), and the 3'/5' ratio for actin was 1.69 (SD 0.36). The spike controls BioB, BioDN, and CreX were present on all arrays. Further quality control was evaluated by the normalized unscaled standard errors (NUSE) for each microarray. One of the EIB^-^ post-exercise microarrays was excluded because of an unacceptably high normalized unscaled standard errors (NUSE) metric as well as failure to meet the manufacturer's quality control guidelines for housekeeping control probe signals which failed the threshold test.

**Differential Gene Expression Between Asthmatic Phenotypes**

Gene expression in airway cells was analyzed between the two phenotypically distinct groups of asthmatics at baseline, and between the groups after exercise challenge. We used two methods to identify genes with the most reproducible differential expression between the groups. In the first method, we identified genes that were differentially expressed in the initial cohort (first 3 subjects in each group) and in the replication cohort (last three subjects in each group), and narrowed the list of genes to those with Log_2_ [fold change] (Log_2_FC) ≥ 1.0 and *P* ≤ 0.05 in both cohorts. In the baseline comparison, there were 94 probe sets, representing 78 genes that were differentially expressed (50 increased and 28 decreased) in the initial cohort, and 64 probe sets, representing 53 genes that were differentially expressed (28 increased and 25 decreased) in the replication cohort, including one gene with reproducible differential gene expression in both cohorts. In the post-exercise comparison, there were 138 probe sets, representing 119 genes that were differentially expressed (106 increased and 13 decreased) in the initial cohort, and 349 probe sets, representing 304 genes that were differentially expressed (260 increased and 44 decreased) in the replication cohort, including 37 probes representing 30 unique genes with reproducible differential gene expression in both cohorts. The combined statistical significance of these two sets was assessed, and a conservative Bonferroni correction was applied to identify 1 gene with differential expression between the groups at baseline and 19 genes with differential expression between the groups post-exercise (Manuscript Table 2). We also assessed differences between the groups using the combined *P* value for the whole data set (Tables S6-7). Based on a false discovery rate (FDR) of 10%, 28 genes in the post-exercise comparison were differentially expressed, including all 19 meeting the criteria for reproducible differential expression and 9 additional genes including CLCA2, GPR56, PROM1, TMC5, PFN2, IQCG, EFHC1, SERPINB2, and an expressed sequence tag (EST) (AV720803). Differences in the expression of 10 of these genes were confirmed by qPCR using samples from all individuals in each group, demonstrating a high degree of reproducibility by array or qPCR methods (Manuscript Figure 1D).

**Genes Expression Response to Exercise Challenge**

Because the bronchoconstrictor response to exercise challenge is a distinguishing feature of the EIB phenotype, we analyzed the gene expression response to exercise challenge. We identified genes transcriptionally activated by exercise challenge in the initial cohort and replication cohorts of EIB^+^ subjects. There were 28 probe sets, representing 22 genes that were differentially expressed (22 increased) in the initial cohort, and 153 probe sets, representing 136 genes that were differentially expressed (132 increased and 4 decreased) in the replication cohort, including 22 probe sets representing 16 unique genes with reproducible differential expression in both cohorts. After applying a Bonferroni correction, 9 genes had increased expression and no genes had decreased expression following exercise challenge in the EIB^+^ group (Manuscript Table 3). We also assessed changes in gene expression after exercise challenge using the combined *P* value for the EIB^+^ phenotype (Table S8). Based on a FDR of 10%, 8 genes had increased expression following exercise challenge in the EIB^+^ group, including 3 additional genes CLCA2, FCGBP, and CST1 not observed in our initial selection algorithm. We confirmed exercise-induced increases in expression of 7 of these genes (TFF3, TPSAB, CPA3, KLK11, TSPAN8, AGR2, and SLPI) by qPCR (Manuscript Figure 1G). In the EIB^-^ group, no gene met either criterion for change in expression in response to exercise challenge.

To visualize the differences in the pattern of gene expression, differences in gene expression between the groups at baseline were plotted against the change in gene expression with exercise challenge in the EIB^+^ group (Manuscript Figure 1F). Only transglutaminase 2 (TGM2) was differentially expressed at baseline, while other genes including the mast cell proteases were not differentially expressed at baseline, but had a marked increase following exercise challenge. Other genes such as trefoil factor 3 (TFF3) had modest increase in expression at baseline and further increase after exercise challenge.

We also evaluated in the change in gene expression in response to challenge in the EIB^+^ group relative to the change in expression in the EIB^-^ group (i.e. interaction effect, Table S9) and found that 7 genes TPSB2, CLCA1, FCGBP, CST1, TPSAB1, CPA3, and TFF3 demonstrated increased relative expression based on the reproducibility criteria, and an additional gene CLCA2 based on the FDR.

**Supporting Information References**

1. Hallstrand TS, Chi EY, Singer AG, Gelb MH, Henderson WR, Jr. (2007) Secreted phospholipase A_2_ group X overexpression in asthma and bronchial hyperresponsiveness. Am J Respir Crit Care Med 176: 1072-1078.

2. Hallstrand TS, Martin DP, Hummel JP, Williams BL, Logerfo JP (2009) Initial Test of the Seattle Asthma Severity and Control Questionnaire (SASCQ): A Multidimensional Assessment of Asthma Severity and Control. Ann Allergy Asthma Immunol in press.

3. Miller MR, Hankinson J, Brusasco V, Burgos F, Casaburi R, et al. (2005) Standardisation of spirometry. Eur Respir J 26: 319-338.

4. Crapo RO, Casaburi R, Coates AL, Enright PL, Hankinson JL, et al. (2000) Guidelines for methacholine and exercise challenge testing-1999. Am J Respir Crit Care Med 161: 309-329.

5. Hallstrand TS, Moody MW, Wurfel MM, Schwartz LB, Henderson WR, Jr., et al. (2005) Inflammatory basis of exercise-induced bronchoconstriction. Am J Respir Crit Care Med 172: 679-686.

6. Wurfel MM, Park WY, Radella F, Ruzinski J, Sandstrom A, et al. (2005) Identification of high and low responders to lipopolysaccharide in normal subjects: an unbiased approach to identify modulators of innate immunity. J Immunol 175: 2570-2578.

7. Touaibia M, Djimde A, Cao F, Boilard E, Bezzine S, et al. (2007) Inhibition of secreted phospholipase A_2_. 4-glycerol derivatives of 4,5-dihydro-3-(4-tetradecyloxybenzyl)-1,2,4-4H-oxadiazol-5-one with broad activities. J Med Chem 50: 1618-1626.

8. Degousee N, Ghomashchi F, Stefanski E, Singer A, Smart BP, et al. (2002) Groups IV, V, and X phospholipases A_2_s in human neutrophils: role in eicosanoid production and gram-negative bacterial phospholipid hydrolysis. J Biol Chem 277: 5061-5073.

9. Folk JE, Cole PW (1966) Transglutaminase: mechanistic features of the active site as determined by kinetic and inhibitor studies. Biochim Biophys Acta 122: 244-264.

10. Leff JA, Busse WW, Pearlman D, Bronsky EA, Kemp J, et al. (1998) Montelukast, a leukotriene-receptor antagonist, for the treatment of mild asthma and exercise-induced bronchoconstriction. N Engl J Med 339: 147-152.

11. Fahy JV, Boushey HA, Lazarus SC, Mauger EA, Cherniack RM, et al. (2001) Safety and reproducibility of sputum induction in asthmatic subjects in a multicenter study. Am J Respir Crit Care Med 163: 1470-1475.

12. Gentleman RC, Carey VJ, Bates DM, Bolstad B, Dettling M, et al. (2004) Bioconductor: open software development for computational biology and bioinformatics. Genome Biol 5: R80.

13. Smyth GK (2004) Linear models and empirical bayes methods for assessing differential expression in microarray experiments. Stat Appl Genet Mol Biol 3: Article3.

14. Hess A, Iyer H (2007) Fisher's combined p-value for detecting differentially expressed genes using Affymetrix expression arrays. BMC Genomics 8: 96.

15. Tusher VG, Tibshirani R, Chu G (2001) Significance analysis of microarrays applied to the ionizing radiation response. Proc Natl Acad Sci U S A 98: 5116-5121.

16. Efthimiadis A, Spanevello A, Hamid Q, Kelly MM, Linden M, et al. (2002) Methods of sputum processing for cell counts, immunocytochemistry and in situ hybridisation. Eur Respir J Suppl 37: 19s-23s.

17. Hallstrand TS, Moody MW, Aitken ML, Henderson WR, Jr. (2005) Airway immunopathology of asthma with exercise-induced bronchoconstriction. J Allergy Clin Immunol 116: 586-593.
