# Supplementary material for: Transglutaminase 2, a Novel Regulator of Eicosanoid Production in Asthma Revealed by Genome-Wide Expression Profiling of Distinct Asthma Phenotypes
Source: PLoS One. 2010 Jan 5;5(1):e8583. doi: 10.1371/journal.pone.0008583 (PMC2797392; doi:10.1371/journal.pone.0008583)
Supplement: Table S1 — Reproducibility of cellular components of induced sputum on 2 separate visits (0.03 MB DOC) [file pone.0008583.s005.doc]

| **Table S1. Reproducibility of cellular components of induced sputum on 2 separate visits** | |
| --- | --- |
| Variable | Concordance Correlation Coefficient (95% CI) |
| Lower airway cells/ml^*, †^ | 0.74 (0.48, 0.99) |
| Eosinophil % | 0.68 (0.38, 0.98) |
| Lymphocyte % | 0.42 (-0.03, 0.87) |
| Macrophage % | 0.80 (0.61, 0.99) |
| Neutrophil % | 0.68 (0.39, 0.98) |
| Epithelial Cell % | 0.78 (0.56, 0.99) |

^*^ The concordance correlation estimates are based on log transformed data.

^†^ “Lower airway cells” refers to inflammatory cells and columnar epithelial cells, excluding non-squamous epithelial cells
