# Supplementary material for: Transglutaminase 2, a Novel Regulator of Eicosanoid Production in Asthma Revealed by Genome-Wide Expression Profiling of Distinct Asthma Phenotypes
Source: PLoS One. 2010 Jan 5;5(1):e8583. doi: 10.1371/journal.pone.0008583 (PMC2797392; doi:10.1371/journal.pone.0008583)
Supplement: Table S2 — Baseline differences in selected induced sputum* (0.05 MB DOC) [file pone.0008583.s006.doc]

| Table S2. Baseline differences in selected induced sputum^*^ | | | | | |
| --- | --- | --- | --- | --- | --- |
|  | **Asthma** | | | |  |
|  | EIB^+^ (*n*=7) | | **EIB^-^ (*n*=7)** | | ***P* value^†^** |
|  | **Percentage** | | | |  |
| Eosinophils^‡^ | 1.83 | (0.61-7.67) | 0.32 | (0.11-1.14) | 0.026 |
| Lymphocytes^‡^ | 1.56 | (1.04-2.25) | 1.25 | (0.78-2.91) | 0.902 |
| Macrophages^‡^ | 43.17 | (36.16-61.32) | 44.95 | (35.74-64.58) | 1.000 |
| Neutrophils^‡^ | 26.21 | (21.64-38.64) | 28.64 | (21.13-49.30) | 0.805 |
| Columnar Epithelial cells^‡^ | 6.35 | (4.50-16.87) | 5.78 | (4.42-17.54) | 0.710 |
| Squamous Epithelial cells^§^ | 8.70 | (5.50-27.94) | 5.12 | (3.88-14.55) | 0.209 |
| Other Cells^¶^ | 5.27 | (3.62-27.94) | 4.93 | (2.20-7.36) | 0.902 |
|  | | | | | |
|  | **Concentration (x 10^4^)** | | | |  |
| Eosinophils^‡^ | 3.27 | (1.03-9.68) | 1.28 | (0.76-4.64) | 0.259 |
| Lymphocytes^‡^ | 3.12 | (1.83-4.43) | 6.11 | (0.76-18.50) | 0.318 |
| Macrophages^‡^ | 157.92 | (30.20-173.78) | 240.58 | (86.66-545.92) | 0.318 |
| Neutrophils^‡^ | 69.79 | (41.55-126.39) | 102.48 | (59.08-274.67) | 0.318 |
| Columnar Epithelial cells^‡^ | 19.51 | (3.12-28.44) | 21.05 | (20.12-44.95) | 0.318 |
| Squamous Epithelial Cells^§^ | 18.95 | (12.41-21.75) | 18.23 | (17.40-39.57) | 0.710 |
| Other Cells^¶^ | 10.68 | (3.39-36.44) | 16.32 | (6.50-39.70) | 0.456 |

^*^ Based on the average of 2 separate induced sputums collected on average 6.7 days apart. Data expressed as median (interquartile range)

^†^ Mann-Whitney U test

^‡^ Inflammatory cells and columnar epithelial cells expressed as percent of non-squamous epithelial cells

^§^ Squamous epithelial cells expressed as the percentage of total cells

^¶^ Cells that could not be classified
