# Supplementary material for: Transglutaminase 2, a Novel Regulator of Eicosanoid Production in Asthma Revealed by Genome-Wide Expression Profiling of Distinct Asthma Phenotypes
Source: PLoS One. 2010 Jan 5;5(1):e8583. doi: 10.1371/journal.pone.0008583 (PMC2797392; doi:10.1371/journal.pone.0008583)
Supplement: Table S3 — Post-exercise Differences in Selected Induced Sputum* (0.05 MB DOC) [file pone.0008583.s007.doc]

| Table S3. Post-exercise Differences in Selected Induced Sputum^*^ | | | | | |
| --- | --- | --- | --- | --- | --- |
|  | **Asthma** | | | |  |
|  | EIB^+^ (n=7) | | **EIB^-^ (n=7)** | | **P value^†^** |
|  | **Percentage** | | | |  |
| Eosinophils^‡^ | 1.98 | (0.97-11.32) | 0.10 | (0.10-0.18) | 0.017 |
| Lymphocytes^‡^ | 1.55 | (0.66-2.10) | 1.12 | (0.63-1.92) | 0.620 |
| Macrophages^‡^ | 32.80 | (22.47-50.73) | 44.13 | (19.54-49.16) | 0.902 |
| Neutrophils^‡^ | 26.74 | (20.34-43.83) | 33.09 | (12.66-60.29) | 0.535 |
| Columnar Epithelial cells^‡^ | 14.22 | (8.53-40.56) | 18.33 | (6.71-36.71) | 0.805 |
| Squamous Epithelial cells^§^ | 9.74 | (6.58-15.09) | 7.67 | (3.80-10.26) | 0.259 |
| Other Cells^¶^ | 6.78 | (2.10-7.71) | 2.61 | (1.08-7.91) | 0.805 |
|  | | | | | |
|  | **Concentration (x 10^4^)** | | | |  |
| Eosinophils^‡^ | 7.56 | (2.47-48.51) | 0.48 | (0.22-0.94) | 0.026 |
| Lymphocytes^‡^ | 2.81 | (2.44-10.10) | 3.03 | (1.53-9.89) | 0.902 |
| Macrophages^‡^ | 84.63 | (58.85-208.28) | 108.85 | (56.26-223.42) | 0.805 |
| Neutrophils^‡^ | 103.44 | (45.75-137.60) | 119.00 | (44.84-319.71) | 0.535 |
| Columnar Epithelial cells^‡^ | 53.55 | (15.57-228.81) | 100.61 | (6.54-176.58) | 0.805 |
| Squamous Epithelial cells^§^ | 31.18 | (15.53-39.69) | 22.18 | (17.46-32.29) | 0.710 |
| Other Cells^¶^ | 17.29 | (2.44-30.22) | 8.88 | (3.88-38.05) | 0.902 |

^*^ Induced sputum collected 30 min after the conclusion of exercise challenge. Data expressed as median (interquartile range)

^†^ Mann-Whitney U test

^‡^ Inflammatory cells and columnar epithelial cells expressed as percent of non-squamous epithelial cells

^§^ Squamous epithelial cells expressed as the percentage of total cells

^¶^ Cells that could not be classified
