# Supplementary material for: Transglutaminase 2, a Novel Regulator of Eicosanoid Production in Asthma Revealed by Genome-Wide Expression Profiling of Distinct Asthma Phenotypes
Source: PLoS One. 2010 Jan 5;5(1):e8583. doi: 10.1371/journal.pone.0008583 (PMC2797392; doi:10.1371/journal.pone.0008583)
Supplement: Table S4 — Regression analysis of differences in selected induced sputum at baseline* (0.04 MB DOC) [file pone.0008583.s008.doc]

| **Table S4. Regression analysis of differences in selected induced sputum at baseline^*^** | | | | | | |
| --- | --- | --- | --- | --- | --- | --- |
|  | **Geometric Mean (x10^4^)** | | **Unadjusted** | | **Methacholine**^†^ | |
|  | **EIB^+^** | **EIB^-^** | **β^§^** | ***P* value** | **β** | ***P* value** |
| Eosinophils | 3.43 | 1.38 | 0.395 | 0.223 | 0.421 | 0.369 |
| Lymphocytes | 2.43 | 5.27 | 0.336 | 0.343 | 0.112 | 0.822 |
| Macrophages | 83.60 | 172.54 | 0.315 | 0.259 | 0.204 | 0.607 |
| Neutrophils | 51.27 | 131.92 | 0.410 | 0.242 | 0.431 | 0.395 |
| Columnar Epithelial Cells | 9.64 | 20.46 | 0.327 | 0.363 | 0.585 | 0.260 |
| Other Cells^¶^ | 8.85 | 16.03 | 0.292 | 0.394 | 0.304 | 0.490 |

* Based on the average of 2 separate induced sputums collected on average 6.7 days apart. Data expressed as geometric mean. The comparison between the groups was made using a regression analysis of the log-transformed values.

^†^ Adjusted for the log methacholine PC_20_ in the regression model

^§^ The β coefficient represents the mean difference in log-transformed values

^¶^ Cells that could not be classified
