# Supplementary material for: Transglutaminase 2, a Novel Regulator of Eicosanoid Production in Asthma Revealed by Genome-Wide Expression Profiling of Distinct Asthma Phenotypes
Source: PLoS One. 2010 Jan 5;5(1):e8583. doi: 10.1371/journal.pone.0008583 (PMC2797392; doi:10.1371/journal.pone.0008583)
Supplement: Table S5 — Regression analysis of differences in selected induced sputum post-exercise* (0.04 MB DOC) [file pone.0008583.s009.doc]

| **Table S5. Regression analysis of differences in selected induced sputum post-exercise^*^** | | | | | | |
| --- | --- | --- | --- | --- | --- | --- |
|  | **Geometric Mean (x10^4^)** | | **Unadjusted** | | **Methacholine**^†^ | |
|  | **EIB^+^** | **EIB^-^** | **β^§^** | ***P* value** | **β** | ***P* value** |
| Eosinophils | 7.47 | 6.02 | 1.094 | 0.013 | 1.186 | 0.052 |
| Lymphocytes | 3.46 | 3.20 | 0.034 | 0.891 | 0.305 | 0.378 |
| Macrophages | 108.04 | 122.72 | 0.055 | 0.740 | 0.010 | 0.967 |
| Neutrophils | 80.41 | 117.95 | 0.166 | 0.421 | 0.302 | 0.314 |
| Columnar Epithelial cells | 44.35 | 51.11 | 0.062 | 0.875 | 0.525 | 0.335 |
| Other Cells^¶^ | 9.93 | 11.37 | 0.059 | 0.858 | 0.798 | 0.043 |

* Induced sputum collected 30 min after the conclusion of exercise challenge. Data expressed as geometric mean. The comparison between the groups was made using a regression analysis of the log-transformed values.

^†^ Adjusted for the log methacholine PC_20_ in the regression model

^§^ The β coefficient represents the mean difference in log-transformed values

^¶^ Cells that could not be classified
