# Supplementary material for: Transglutaminase 2, a Novel Regulator of Eicosanoid Production in Asthma Revealed by Genome-Wide Expression Profiling of Distinct Asthma Phenotypes
Source: PLoS One. 2010 Jan 5;5(1):e8583. doi: 10.1371/journal.pone.0008583 (PMC2797392; doi:10.1371/journal.pone.0008583)
Supplement: Table S6 — Genes with increased expression in EIB+ group relative to EIB- group at baseline (Log2FC>1, P<0.05) (0.04 MB DOC) [file pone.0008583.s010.doc]

| **Table S6. Genes with increased expression in EIB^+^ group relative to EIB^-^ group at baseline (Log_2_FC > 1, p < 0.05)** | | | | | |
| --- | --- | --- | --- | --- | --- |
| GenBank | Log_2_FC | P value | FDR | Symbol | Description |
| BC003551 | 2.04 | 0.0002 | 0.590 | TGM2 | Transglutaminase 2 |
| NM_003226 | 1.84 | 0.0024 | 0.618 | TFF3 | Trefoil factor 3 (intestinal) |
| NM_003064 | 1.83 | 0.0090 | 0.618 | SLPI | Secretory leukocyte peptidase inhibitor |
| BE675337 | 1.67 | 0.0159 | 0.618 | GSN | Gelsolin (amyloidosis, Finnish type) |
| AF088867 | 2.34 | 0.0168 | 0.618 | AGR2 | Anterior gradient homolog 2 (*Xenopus laevis*) |
| NM_001828 | 3.22 | 0.0327 | 0.618 | CLC | Charcot-Leyden crystal protein |
| BG166705 | 2.14 | 0.0402 | 0.618 | CXCL5 | Chemokine (C-X-C motif) ligand 5 |
| N74607 | 1.11 | 0.0467 | 0.618 | AQP3 | Aquaporin 3 (Gill blood group) |
| NM_004616 | 1.62 | 0.0471 | 0.618 | TSPAN8 | Tetraspanin 8 |
| BF055462 | 1.85 | 0.0520 | 0.618 | THBS1 | Thrombospondin 1 |
| AK000168 | 2.30 | 0.0520 | 0.618 | CD24 | CD24 |
