# Supplementary material for: Transglutaminase 2, a Novel Regulator of Eicosanoid Production in Asthma Revealed by Genome-Wide Expression Profiling of Distinct Asthma Phenotypes
Source: PLoS One. 2010 Jan 5;5(1):e8583. doi: 10.1371/journal.pone.0008583 (PMC2797392; doi:10.1371/journal.pone.0008583)
Supplement: Table S7 — Genes with differential expression in EIB+ group relative to EIB- group post exercise (Log2FC>1, P<0.05) (0.13 MB DOC) [file pone.0008583.s011.doc]

| **Table S7. Genes with differential expression in EIB^+^ group relative to EIB^-^ group post exercise (Log_2_FC > 1, p<0.05)** | | | | | |
| --- | --- | --- | --- | --- | --- |
| GenBank | Log_2_FC | P value | FDR | Symbol | Description |
| BF003134 | 2.37 | 0.000000 | 0.000 | CLCA2 | Chloride channel, Ca^2+^-activated, member 2 |
| NM_003226 | 4.80 | 0.000000 | 0.000 | TFF3 | Trefoil factor 3 |
| AF127036 | 5.23 | 0.000000 | 0.000 | CLCA1 | Chloride channel, Ca^2+^-activated, member 1 |
| NM_024164 | 3.50 | 0.000000 | 0.001 | TPSB2 | Tryptase β2 |
| NM_001898 | 3.87 | 0.000001 | 0.001 | CST1 | Cystatin SN |
| NM_003890 | 2.85 | 0.000001 | 0.002 | FCGBP | Fc fragment of IgG-binding protein |
| NM_001870 | 4.70 | 0.000005 | 0.006 | CPA3 | Carboxypeptidase A3 (mast cell) |
| AF206667 | 3.94 | 0.000006 | 0.006 | TPSAB1 | Tryptase α/β1 |
| NM_006853 | 2.12 | 0.000009 | 0.007 | KLK11 | Kallikrein-related peptidase 11 |
| NM_004616 | 4.25 | 0.000015 | 0.011 | TSPAN8 | Tetraspanin 8 |
| AF088867 | 4.69 | 0.000036 | 0.020 | AGR2 | Anterior gradient homolog 2 (*Xenopus laevis*) |
| AI743792 | 2.06 | 0.000037 | 0.020 | ST6GAL1 | ST6 β-galactosamide α-2,6-sialyltranferase 1 |
| AV720803 | 2.67 | 0.000118 | 0.046 |  |  |
| NM_003064 | 3.03 | 0.000137 | 0.049 | SLPI | Secretory leukocyte peptidase inhibitor |
| AI521646 | 4.76 | 0.000157 | 0.051 | MUC5AC | Mucin 5AC, oligomeric mucus/gel-forming |
| AL554008 | 2.02 | 0.000160 | 0.051 | GPR56 | G protein-coupled receptor 56 |
| NM_006017 | 3.91 | 0.000189 | 0.055 | PROM1 | Prominin 1 |
| NM_016140 | 3.02 | 0.000201 | 0.056 | CGI-38 | Brain-specific protein |
| NM_015180 | 1.73 | 0.000205 | 0.056 | SYNE2 | Spectrin repeat containing nuclear envelope 2 |
| NM_015717 | 1.93 | 0.000290 | 0.065 | CD207 | CD207, langerin |
| NM_001828 | 5.34 | 0.000290 | 0.065 | CLC | Charcot-Leyden crystal protein |
| NM_024780 | 2.30 | 0.000378 | 0.080 | TMC5 | Transmembrane channel-like 5 |
| NM_002628 | 3.12 | 0.000390 | 0.081 | PFN2 | Profilin 2 |
| NM_025111 | 2.02 | 0.000490 | 0.094 | IQCG | IQ motif containing G |
| AK000168 | 4.03 | 0.000583 | 0.100 | CD24 | CD24 |
| AF133425 | 3.97 | 0.000703 | 0.109 | TSPAN1 | Tetraspanin 1 |
| NM_018100 | 2.89 | 0.000759 | 0.110 | EFHC1 | EF-hand domain (C-terminal) containing 1 |
| NM_002575 | 3.06 | 0.000969 | 0.121 | SERPINB2 | serpin peptidase inhibitor, clade B, member 2 |
| NM_000846 | 2.20 | 0.001417 | 0.149 | GSTA2 | Glutathione S-transferase A2 |
| BC005238 | 3.32 | 0.001424 | 0.149 | FXYD3 | FXYD domain ion transport regulator 3 |
| NM_001553 | 2.55 | 0.001509 | 0.155 | IGFBP7 | Insulin-like growth factor binding protein 7 |
| NM_001306 | 1.86 | 0.001536 | 0.156 | CLDN3 | Claudin 3 |
| NM_003551 | 2.29 | 0.001569 | 0.157 | NME5 | Non-metastatic cells 5 protein |
| NM_019114 | 2.10 | 0.001644 | 0.160 | EPB41L4B | Erythrocyte membrane protein 4.1 like 4B |
| NM_001140 | 3.66 | 0.001684 | 0.160 | ALOX15 | Arachidonate 15-lipoxygenase |
| AF079363 | 2.70 | 0.001799 | 0.166 | SPAG6 | Sperm-associated antigen 6 |
| M16768 | 1.36 | 0.002205 | 0.190 | TRGV9 | T cell-receptor gamma variable 9 |
| NM_002615 | 1.94 | 0.002396 | 0.198 | SERPINF1 | Serpin-peptidase inhibitor, clade F, member 1 |
| J02871 | 4.31 | 0.002430 | 0.198 | CYP4B1 | Cytochrome P450, family 4, subfamily B1 |
| L07555 | 2.40 | 0.002433 | 0.198 | CD69 | CD69 |
| NM_001889 | 1.09 | 0.003051 | 0.226 | CRYZ | Crystallin, zeta (quinone reductase) |
| M25915 | 2.98 | 0.003148 | 0.226 | CLU | Clusterin |
| BC005008 | 2.58 | 0.003475 | 0.242 | CEACAM6 | CEA-related cell adhesion molecule 6 |
| AK025298 | 2.45 | 0.004740 | 0.270 | AUTS2 | Autism susceptibility candidate 2 |
| NM_006103 | 2.70 | 0.005073 | 0.274 | WFDC2 | WAP four-disulfide core domain 2 |
| NM_005410 | 1.89 | 0.005104 | 0.274 | SEPP1 | Selenoprotein P, plasma, 1 |
| AF053453 | 1.56 | 0.007084 | 0.316 | TSPAN6 | Tetraspanin 6 |
| NM_000224 | 2.98 | 0.007359 | 0.318 | KRT18 | Keratin 18 |
| BF063271 | 1.70 | 0.007794 | 0.319 | GALNT3 | Gal N-acetylgalactosaminyltransferase 3 |
| NM_025257 | 2.74 | 0.007822 | 0.319 | SLC44A4 | Solute carrier family 44, member 4 |
| BC003551 | 1.34 | 0.007923 | 0.319 | TGM2 | Transglutaminase 2 |
| NM_004944 | 1.89 | 0.008089 | 0.319 | DNASE1L3 | Deoxyribonuclease I-like 3 |
| NM_001263 | 2.10 | 0.009264 | 0.339 | CDS1 | CDP-diacylglycerol synthase 1 |
| N74607 | 1.45 | 0.009438 | 0.341 | AQP3 | Aquaporin 3 (Gill blood group) |
| NM_001954 | 1.84 | 0.012177 | 0.365 | DDR1 | Discoidin domain receptor family, member 1 |
| NM_003570 | 1.40 | 0.014226 | 0.382 | CMAH | CMP-N-acetylneuraminate monooxygenase |
| NM_003059 | 1.32 | 0.014421 | 0.382 | SLC22A4 | Solute carrier family 22, member 4 |
| BE968833 | 1.62 | 0.014576 | 0.383 | SPTBN1 | Spectrin β non-erythrocytic 1 |
| NM_001765 | 2.77 | 0.016206 | 0.390 | CD1C | CD1c molecule |
| NM_003944 | 2.23 | 0.016851 | 0.395 | SELENBP1 | Selenium binding protein 1 |
| U19556 | 3.82 | 0.020115 | 0.412 | SERPINB3 | Serpin peptidase inhibitor, clade B, member 3 |
| NM_004165 | 1.85 | 0.020956 | 0.415 | RRAD | Ras-related associated with diabetes |
| M92934 | 2.05 | 0.021162 | 0.415 | CTGF | Connective tissue growth factor |
| NM_004363 | 2.25 | 0.023850 | 0.431 | CEACAM5 | CEA-related cell adhesion molecule 5 |
| J04152 | 3.03 | 0.025494 | 0.438 | TACSTD2 | Tumor-associated calcium signal transducer 2 |
| U73844 | 1.58 | 0.025902 | 0.438 | ELF3 | E74-like factor 3 |
| NM_030915 | 1.23 | 0.026963 | 0.438 | LBH | Limb bud and heart development homolog |
| NM_020672 | 1.58 | 0.027540 | 0.441 | S100A14 | S100 calcium binding protein A14 |
| NM_001837 | 1.74 | 0.029078 | 0.444 | CCR3 | Chemokine (C-C motif) receptor 3 |
| U76549 | 1.59 | 0.033467 | 0.453 | KRT8 | Keratin 8 |
| NM_001038 | 1.72 | 0.033491 | 0.453 | SCNN1A | Sodium channel, nonvoltage-gated 1 α |
| NM_007173 | 2.21 | 0.033697 | 0.453 | PRSS23 | Protease, serine, 23 |
| U19557 | 2.19 | 0.034085 | 0.453 | SERPINB4 | Serpin peptidase inhibitor, clade B, member 4 |
| AI610869 | 1.20 | 0.034931 | 0.453 | MUC1 | Mucin 1, cell surface associated |
| NM_002443 | 2.99 | 0.037301 | 0.456 | MSMB | β-Microseminoprotein |
| AV726673 | 2.45 | 0.040217 | 0.459 | THBS1 | Thrombospondin 1 |
| NM_005173 | 1.71 | 0.041504 | 0.459 | ATP2A3 | ATPase, Ca^2+^-transporting, ubiquitous |
| NM_007231 | 2.56 | 0.042037 | 0.459 | SLC6A14 | Solute carrier family 6, member 14 |
| NM_004360 | 2.73 | 0.046611 | 0.463 | CDH1 | Cadherin 1, type 1, E-cadherin (epithelial) |
|  |  |  |  |  |  |
| NM_000064 | -1.56 | 0.045088 | 0.618 | C3 | Complement component 3 |
