# Supplementary material for: Transglutaminase 2, a Novel Regulator of Eicosanoid Production in Asthma Revealed by Genome-Wide Expression Profiling of Distinct Asthma Phenotypes
Source: PLoS One. 2010 Jan 5;5(1):e8583. doi: 10.1371/journal.pone.0008583 (PMC2797392; doi:10.1371/journal.pone.0008583)
Supplement: Table S8 — Genes with change in expression after exercise challenge in the EIB+ group (Log2FC>1, P<0.05) (0.08 MB DOC) [file pone.0008583.s012.doc]

| **Table S8. Genes with change in expression after exercise challenge in the EIB^+^ group (Log_2_FC > 1, *P* < 0.05)** | | | | | | | | |
| --- | --- | --- | --- | --- | --- | --- | --- | --- |
| GenBank | Log_2_FC | P value | | FDR | Symbol | | Description | |
| BF003134 | 2.56 | 0.000000 | 0.000 | | | CLCA2 | | Chloride channel, Ca^2+^-activated, member 2 |
| NM_024164 | 3.69 | 0.000000 | 0.001 | | | TPSB2 | | Tryptase β2 |
| AF127036 | 4.46 | 0.000001 | 0.006 | | | CLCA1 | | Chloride channel, Ca^2+^-activated, member 1 |
| NM_003226 | 3.48 | 0.000003 | 0.011 | | | TFF3 | | Trefoil factor 3 (intestinal) |
| NM_003890 | 2.27 | 0.000005 | 0.016 | | | FCGBP | | Fc fragment of IgG-binding protein |
| NM_003294 | 3.63 | 0.000009 | 0.020 | | | TPSAB1 | | Tryptase α/β1 |
| NM_001870 | 4.46 | 0.000010 | 0.020 | | | CPA3 | | Carboxypeptidase A3 (mast cell) |
| NM_001898 | 2.95 | 0.000013 | 0.023 | | | CST1 | | Cystatin SN |
| NM_006853 | 1.58 | 0.000239 | 0.231 | | | KLK11 | | Kallikrein-related peptidase 11 |
| NM_015717 | 1.90 | 0.000334 | 0.297 | | | CD207 | | CD207 molecule, langerin |
| AI521646 | 4.31 | 0.000413 | 0.328 | | | MUC5AC | | Mucin 5AC, oligomeric mucus/gel-forming |
| AV720803 | 2.36 | 0.000413 | 0.328 | | | EST^*^ | | EST cDNA clone GLCCMA04 |
| NM_016140 | 2.74 | 0.000581 | 0.403 | | | CGI-38 | | Brain specific protein |
| NM_004616 | 2.96 | 0.000713 | 0.480 | | | TSPAN8 | | Tetraspanin 8 |
| AL554008 | 1.60 | 0.001803 | 0.791 | | | GPR56 | | G protein-coupled receptor 56 |
| NM_006017 | 3.01 | 0.002010 | 0.827 | | | PROM1 | | Prominin 1 |
| NM_000846 | 2.17 | 0.002099 | 0.848 | | | GSTA2 | | Glutathione S-transferase A2 |
| NM_006732 | 1.07 | 0.002253 | 0.881 | | | FOSB | | FBJ osteosarcoma viral oncogene homolog B |
| NM_004430 | 1.41 | 0.002535 | 0.954 | | | EGR3 | | Early growth response 3 |
| NM_015180 | 1.29 | 0.003120 | 1.000 | | | SYNE2 | | spectrin repeat containing, nuclear envelope 2 |
| NM_014399 | 1.67 | 0.003569 | 1.000 | | | TSPAN13 | | Tetraspanin 13 |
| AF079363 | 2.45 | 0.003704 | 1.000 | | | SPAG6 | | Sperm-associated antigen 6 |
| U34690 | 1.27 | 0.004355 | 1.000 | | | CORO1A | | Coronin, actin binding protein, 1A |
| NM_002628 | 2.37 | 0.004447 | 1.000 | | | PFN2 | | Profilin 2 |
| NM_018100 | 2.24 | 0.005614 | 1.000 | | | EFHC1 | | EF-hand domain (C-terminal) containing 1 |
| M25915 | 2.12 | 0.008245 | 1.000 | | | CLU | | Clusterin |
| NM_003059 | 1.46 | 0.008559 | 1.000 | | | SLC22A4 | | Solute carrier family 22, member 4 |
| NM_030915 | 1.48 | 0.009458 | 1.000 | | | LBH | | limb bud and heart development homolog |
| AB002384 | 1.68 | 0.009899 | 1.000 | | | C6orf32 | | Chromosome 6 open reading frame 32 |
| NM_002309 | 1.34 | 0.009943 | 1.000 | | | LIF | | Leukemia inhibitory factor |
| NM_005410 | 1.61 | 0.010698 | 1.000 | | | SEPP1 | | Selenoprotein P, plasma, 1 |
| AV733950 | 2.00 | 0.011193 | 1.000 | | | EGR1 | | Early growth response 1 |
| NM_001263 | 2.05 | 0.012045 | 1.000 | | | CDS1 | | CDP-diacylglycerol synthase 1 |
| NM_004165 | 2.03 | 0.012459 | 1.000 | | | RRAD | | Ras-related associated with diabetes |
| NM_019114 | 1.59 | 0.013552 | 1.000 | | | EPB41L4B | | Erythrocyte membrane protein 4.1 like 4B |
| AF088867 | 2.35 | 0.014785 | 1.000 | | | AGR2 | | Anterior gradient homolog 2 (*Xenopus laevis*) |
| BF063271 | 1.55 | 0.015962 | 1.000 | | | GALNT3 | | Gal N-acetylgalactosaminyltransferase 3 |
| NM_003064 | 1.59 | 0.020176 | 1.000 | | | SLPI | | Secretory leukocyte peptidase inhibitor |
| NM_005173 | 1.67 | 0.023775 | 1.000 | | | ATP2A3 | | ATPase, Ca^2+^-transporting, ubiquitous |
| M58664 | 1.63 | 0.024834 | 1.000 | | | CD24 | | CD24 molecule |
| NM_001828 | 2.45 | 0.026187 | 1.000 | | | CLC | | Charcot-Leyden crystal protein |
| NM_001837 | 1.14 | 0.028772 | 1.000 | | | CCR3 | | Chemokine (C-C motif) receptor 3 |
| AF133425 | 2.35 | 0.031933 | 1.000 | | | TSPAN1 | | Tetraspanin 1 |
| AF003934 | 1.19 | 0.038627 | 1.000 | | | GDF15 | | Growth differentiation factor 15 |
| AI935096 | 1.07 | 0.043293 | 1.000 | | | NR4A2 | | Nuclear receptor subfamily 4, group A2 |
| NM_002575 | 1.80 | 0.043416 | 1.000 | | | SERPINB2 | | Serpin peptidase inhibitor, clade B, member 2 |
| J02871 | 2.78 | 0.043940 | 1.000 | | | CYP4B1 | | Cytochrome P450, family 4, subfamily B1 |
| BC005008 | 1.58 | 0.045734 | 1.000 | | | CEACAM6 | | CEA-related cell adhesion molecule 6 |

^*^ Expressed Sequence Tag.
