# Supplementary material for: Transglutaminase 2, a Novel Regulator of Eicosanoid Production in Asthma Revealed by Genome-Wide Expression Profiling of Distinct Asthma Phenotypes
Source: PLoS One. 2010 Jan 5;5(1):e8583. doi: 10.1371/journal.pone.0008583 (PMC2797392; doi:10.1371/journal.pone.0008583)
Supplement: Table S9 — Genes with changes in expression after exercise challenge (post-exercise minus baseline) in the EIB+ group relative to EIB- group (Log2FC>1, P<0.05) (0.06 MB DOC) [file pone.0008583.s013.doc]

| **Table S9. Genes with changes in expression after exercise challenge (post-exercise – baseline) in the EIB^+^ group relative to EIB^-^ group (Log_2_FC > 1, *P* < 0.05)** | | | | | |
| --- | --- | --- | --- | --- | --- |
| GenBank | Log_2_FC | P value | FDR | Symbol | Description |
| BF003134 | 2.44 | 0.000000 | 0.002 | CLCA2 | Chloride channel, Ca^2+^-activated, member 2 |
| NM_024164 | 3.52 | 0.000024 | 0.271 | TPSB2 | tryptase β2 |
| AF127036 | 4.24 | 0.000156 | 0.449 | CLCA1 | Chloride channel, Ca^2+^-activated, member 1 |
| NM_003890 | 2.14 | 0.000221 | 0.546 | FCGBP | Fc fragment of IgG binding protein |
| NM_001898 | 2.96 | 0.000477 | 0.920 | CST1 | Cystatin SN |
| NM_003294 | 3.59 | 0.000662 | 0.920 | TPSAB1 | Tryptase α/β1 |
| NM_001870 | 4.23 | 0.001055 | 1.000 | CPA3 | Carboxypeptidase A3 (mast cell) |
| NM_003226 | 2.96 | 0.001129 | 1.000 | TFF3 | Trefoil factor 3 (intestinal) |
| NM_006853 | 1.53 | 0.009721 | 1.000 | KLK11 | Kallikrein-related peptidase 11 |
| NM_025111 | 1.96 | 0.012709 | 1.000 | IQCG | IQ motif-containing G |
| NM_016140 | 2.64 | 0.013523 | 1.000 | CGI-38 | Brain-specific protein |
| NM_006017 | 3.11 | 0.018326 | 1.000 | PROM1 | Prominin 1 |
| AL554008 | 1.54 | 0.029320 | 1.000 | GPR56 | G protein-coupled receptor 56 |
| NM_015717 | 1.56 | 0.032702 | 1.000 | CD207 | CD207, langerin |
| AF079363 | 2.46 | 0.033356 | 1.000 | SPAG6 | Sperm associated antigen 6 |
| NM_004616 | 2.62 | 0.033713 | 1.000 | TSPAN8 | Tetraspanin 8 |
| NM_014399 | 1.69 | 0.037913 | 1.000 | TSPAN13 | Tetraspanin 13 |
| NM_002628 | 2.37 | 0.046259 | 1.000 | PFN2 | Profilin 2 |
| NM_000846 | 2.04 | 0.047087 | 1.000 | GSTA2 | Glutathione S-transferase A2 |
| AW192795 | 3.74 | 0.048722 | 1.000 | MUC5AC | Mucin 5AC, oligomeric mucus/gel-forming |
| NM_012105 | 1.81 | 0.049197 | 1.000 | BACE2 | β-site APP-cleaving enzyme 2 |
| M25915 | 2.10 | 0.052054 | 1.000 | CLU | Clusterin |
| NM_001263 | 2.27 | 0.052476 | 1.000 | CDS1 | CDP-diacylglycerol synthase 1 |
| NM_002575 | 2.46 | 0.052624 | 1.000 | SERPINB2 | Serpin peptidase inhibitor, clade B, member 2 |
